# The dose–response effect of time between emergency admission and inpatient care on mortality

**DOI:** 10.1038/s41598-023-49090-5

**Published:** 2023-12-14

**Authors:** S. Castaño-Pérez, J. A. Medina García, A. Cabrera de León

**Affiliations:** 1 Internal Medicine Department, Hospital Universitario Nuestra Señora de la Candelaria, Santa Cruz de Tenerife, Spain; 2 Internal Medicine Department, Hospital Quirónsalud Tenerife, Santa Cruz de Tenerife, Spain; 3 Research Unit of Primary Care, Tenerife, Spain; 4 Preventive Medicine and Public Health, Universidad de La Laguna, La Laguna, Spain; 5 Unidad de Investigación, Hospital Universitario NS de La Candelaria, Carretera de El Rosario 145, 38010 Santa Cruz de Tenerife, Canary Islands Spain

**Keywords:** Health care, Risk factors

## Abstract

To analyse mortality associated to emergency admissions on weekends, differentiating whether the patients were admitted to the Internal Medicine department or to the hospital as a whole. Retrospective follow-up study of patients discharged between 2015 and 2019 in: (a) the Internal Medicine department (n = 7656) and (b) the hospital as a whole (n = 83,146). Logistic regression models were fitted to analyse the risk of death, adjusting for age, sex, severity, Charlson index, sepsis, pneumonia, heart failure and day of admission. Cox models were also adjusted for the time from admission until normal inpatient care. There was a significant increase in mortality for patients admitted in weekends with short stays in Internal Medicine (48, 72 and 96 h: OR = 2.50, 1.89 and 1.62, respectively), and hospital-wide (OR = 2.02, 1.41 and 1.13, respectively). The highest risk in weekends occurred on Fridays (stays ≤ 48 h: OR = 3.92 [95% CI 2.06–7.48]), being no significative on Sundays. The risk increased with the time elapsed from admission until the inpatient department took over care (OR = 5.51 [95% CI 1.42–21.40] when this time reached 4 days). In Cox models patients reached HR = 2.74 (1.00–7.54) when the delay was 4 days. Whether it was Internal Medicine or hospital-wide patients, the risk of death associated with emergency admission in WE increased with the time between admission and transfer of care to the inpatient department; consequently, Friday was the day with the highest risk while Sunday lacked a weekend effect. Healthcare systems should correct this serious problem.

## Introduction

Since the late 1980s, suspicions of increased mortality associated with non-working days have been raised and have given rise to the disturbing concept of "weekend effect"^1^. Numerous studies have subsequently shown that differences in patient care during the weekend may have consequences for their health status.

Thus, it has been shown that this difference in care not only affects the weekend, but patients admitted during holiday periods also have increased mortality^2^. There is some research on this problem in Spain^3–6^, although most of the publications on this issue have taken place in the Europe^7–13^ and North America^14–18^, and more recently Asia^19^. Furthermore, most of the studies on the "weekend effect" focus on specific diseases or health problems^11,19–22^ and there are almost no publications on regular patients in Internal Medicine services, in which most of patients suffer from multi-pathological problems and with heterogeneous causes of admission^4^.

Some studies have found that mortality differences observed in patients admitted on weekends are greater in the short term, and as length of stay increases, this "weekend effect" fades^3,4,23–25^. Decreased staffing levels^26–28^, increased severity of illness or delayed procedures^29–31^ have been associated with this increased mortality.

Although most authors agree on the negative impact of this phenomenon, the debate continues, as not all researchers agree that these differences are relevant^6,32–34^. There is little evidence in the field of Internal Medicine, whose patients are highly complex, with pluri-pathological and comorbid problems. Often, emergency physicians do not have the time to comprehensively address the Internal Medicine patient. For this reason we want to discover if admission on the weekend and holidays implies greater mortality according to whether the patients are admitted to the Internal Medicine department or to the hospital as a whole, analysing the length of stay, the severity of illness, and the time from admission to normal inpatient care.

## Methods

### Design and setting

Retrospective follow-up study of patients over 14 years of age admitted to the emergency department of the Hospital Universitario Nuestra Señora de La Candelaria (HUNSC) until their discharge or death, between 2015 and 2019 (n = 83,146); they were not patients scheduled for admission but unplanned emergency admissions. This is a tertiary care hospital with 960 beds, located in Tenerife, Canary Islands, Spain. It serves a population of about 500,000 inhabitants, from which patients are referred to lower-level hospitals, which were excluded as it was not known for sure when death occurred.

### Staffing

The usual medical care of patients admitted to the HUNSC was provided in the morning on working days, with the on-call medical team overseeing emergency care during the evening and night hours, as well as on non-working days. The admission of a patient was decided by a specialist from the department, who must carry out the complete diagnostic and therapeutic procedure even if his or her working hours had ended. When the specialist anticipated that the procedure will require a long time, the evaluation and admission of the patient could be transferred to the on-call team. The medical team on duty in the medical area was in charge of attending to the incidents of patients admitted to the Internal Medicine, Geriatrics, Digestive, Endocrinology, Rheumatology, and Medical Oncology services, as well as admitting patients from these specialities; in the event of multi-pathological medical complications occurring in patients admitted to other specialities, assistance was also provided. The on-call team consisted of 2 specialist physicians and 3 physicians in training throughout the year, except for occasional times of high pressure of care when the number of professionals was exceptionally increased. One of the doctors in training of this on-call team addressed questions/emergencies from the patients already admitted, so the rest of the team was not distracted from the formal emergency room (ER). Nursing staff assigned to the ER during on-call did not undergo changes in WD or WE. Patients admitted to the medical specialties were immediately transferred from the ER to the beds in each department; only patients admitted in MI had to routinely wait, occupying ER beds, until the MI department had beds to which they could be transferred. A longer explanation of how our hospital operates is included as text in the supplemental file.

### Data extraction

Hospital data were obtained from the minimum basic data set (MBDS) of the Spanish Government for hospital discharges in Spain^35^, for the period 2015–2019. Approval of the study was given by the Bioethics Committee of the Nª Sra. de Candelaria University Hospital, with the code CHUNSC_2018_47 and the need for informed consent was waived by the ethical committee of this hospital. All methods were performed in accordance with the relevant guidelines and regulations." The database for this study was produced by the Ministry of Health with aggregated fully anonymized data, so individual written consent by patients is not required in Spain for the use of the BMDS (under the Spanish Organic Law 3/2018 on the protection of personal data and digital rights, which transcribe the European Union General Data Protection Regulation 2016/679).

The data recorded in this registry were obtained excluding patients transferred from other services and hospitals. In addition, for patients admitted to the Internal Medicine department (n = 7656), comorbidity information was extracted on the existence of sepsis, congestive heart failure or pneumonia at the time of admission, and the Charlson index was calculated^36^. For the analysis of the group of patients admitted to Internal Medicine, we excluded those who were moved to the palliative care unit, and also the patients admitted to critical services. For patients admitted in the hospital as a whole, only data from the MBDS (age, sex, year of admission, and working day or holiday) were available; to approximate this group comorbidities, we used the “All Patient Refined Diagnosis Related Groups” (APR-DRG) severity subclasses, which is a validated classification of patients into four levels according to their reason of admission^37^.

### Definitions

Working days (WD) were considered Monday (from 8:00 h), to Friday (up to 15:00 h); patients admitted from Monday to Thursday from 15:00 were transferred daily at 8:00 the next day. Weekends (WE) were defined as Fridays (from 15:01 h), Saturdays, and Sundays (until Monday 7:59), in addition to public holidays, and also the eve of public holidays from 15:01 h. Mortality was calculated and analysed according to: (a) whether the admission occurred on WD or WE; (b) whether it occurred on Friday, Saturday or Sunday; and (c) the time it took for the patients to be attended by the physicians of the department where they were admitted. This time was estimated as the number of days elapsed between the time of admission by the physicians on duty and the transfer of responsibility for care, at the end of the WE, to the physicians of the department where the patient was admitted. The follow-up time for each patient was from admission until hospital discharge or death, since the primary outcome of the study was hospital mortality.

### Statistical analysis

Qualitative variables were summarised with absolute and relative frequencies (%), and continuous variables with the mean ± standard deviation (SD). Bivariate analysis between qualitative variables was performed using Pearson's Chi-square test and between quantitative variables using Student's t-test. The non parametric Kruskal–Wallis test was used when the variables did not approximate the normal distribution.

For the analysis of mortality, logistic regression models (summarised with the OR and its 95% CI) were fitted taking as dependent variable the outcome of death or not, and as independent variables all those mentioned above (age, sex, Charlson index, diagnosis at admission of pneumonia, congestive heart failure, sepsis, year of admission, severity on admission, and WE or WD); Since the length of stay was identified as a confounding variable, associated to the studied exposure and effect (Supplementary Table 1), it was stratified and a model was fitted for each level of this variable (≤ 48, ≤ 72, ≤ 96 and > 96 h). Also Cox proportional risk models, summarised with HR (95% CI), were adjusted including the same independent variables plus the time elapsed (days) until the patient was assessed by the physicians of the department where he/she was admitted, and with death as the dependent variable. Analyses were performed with the SPSS statistical package (version 26, in Spanish) always using bilateral hypotheses.

## Results

A total of 7656 admissions to Internal Medicine were analysed, of which 4296 were admitted on weekdays WD and 3360 on WE. Mean age was similar in both groups (70 years in WD and WE; *P* > 0.05). There were also no differences in sex distribution (WD 53% male vs WE 54%; *P* > 0.05), number of comorbidities (WD 2.64 vs. WE 2.53; *P* > 0.05), or in diagnoses of heart failure, respiratory infection or sepsis, nor in mortality; short stays were significantly more frequent among patients admitted on WD, and there were differences in the distribution of admissions in the different years studied (Table 1). For patients in the hospital as a whole, 83,146 admissions were analysed, of which 48,271 were admitted on WD and 34,875 on WE; also in these patients short stays were significantly more frequent on WD and there were also differences in the distribution of admissions in the different years studied (Table 1). Friday was the day with the highest number of admissions for Internal Medicine while for the hospital as a whole it was Wednesday, with Saturdays and Sundays being the days with the fewest admissions in both groups of patients (Supplementary Table 2). The 10 most common diagnoses for WD and WE emergency admissions are shown in the supplementary Table 3, with no important frequency differences; among them, the highest mortality rate was for pneumonitis due to food inhalation and vomiting, above 20% in WD and WE.

**Table 1:** Distribution of patients, and their mortality, with respect to admission on working days (WD) or weekends (WE).

Internal medicine patients		WD (%) n = 4296	WE (%) n = 3360	*p*
Age x ± SD		69.8 ± 15.9	69.8 ± 16.2	> 0.05
Men		2275 (53.0)	1817 (54.1)	> 0.05
I. Charlson x ± SD		2.6 ± 2.5	2.5 ± 2.5	> 0.05
Sepsis		393 (9.1)	338 (10.1)	> 0.05
Heart failure		1133 (26.4)	825 (24.6)	> 0.05
Pneumonia		374 (8.7)	296 (8.8)	> 0.05
Stay x ± SD		10.7 ± 13.3	10.6 ± 11.1	> 0.05
Stay ≤ 48 h		635 (14.8)	244 (7.3)	< 0.001
Stay ≤ 72 h		904 (21.0)	505 (15.0)	< 0.001
Stay ≤ 96 h		1187 (27.6)	821 (24.4)	< 0.01
Year of discharge	2015	854 (19.9)	554 (16.5)	< 0.05
	2016	762 (17.7)	611 (18.2)	
	2017	908 (21.1)	822 (24.5)	
	2018	844 (19.6)	691 (20.6)	
	2019	928 (21.6)	682 (20.3)	
Total mortality		301 (7.0)	251 (7.5)	> 0.05

Hospital-wide patients		WD (%) n = 48,271	WE (%) n = 34,875	*p*
Age x ± SD		57.0 ± 21.1	57.3 ± 21.5	> 0.05
Men		20,629 (42.7)	15,211 (43.6)	0.01
Severity at admission	1	17,741 (35.5)	12,688 (36.4)	0.041
	2	17,819 (36.9)	12,578 (36.1)	
	3	11,477 (23.8)	8283 (23.8)	
	4	1834 (3.8)	1326 (3.8)	
Stay x ± SD		10.3 ± 19.9	9.9 ± 14.4	< 0.001
Stay ≤ 48 h		12,028 (24.9)	6872 (19.7)	< 0.001
Stay ≤ 72 h		16,334 (33.8)	11,008 (31.6)	< 0.001
Stay ≤ 96 h		20,112 (41.7)	14,276 (40.9)	< 0.05
Year of discharge	2015	9370 (19.4)	5883 (16.9)	< 0.001
	2016	9026 (18.7)	6511 (18.7)	
	2017	10,175 (21.1)	7598 (21.7)	
	2018	9989 (20.7)	7598 (21.8)	
	2019	9711 (20.1)	7327 (21.0)	
Total mortality		2176 (4.5)	1540 (4.4)	> 0.05

Data are presented as mean ± standard deviation (SD) for continuous variables and number (percentage) for categorical variables. The *P* value to the right represents either t-test for continuous data (Kruskal-Wallis test when the variables did not approximate the normal distribution) or chi-square for categorical data.

Stratification of the bivariate analyses according to length of stay (Supplementary Table 4) corroborated the absence of significant differences between WD and WE for Internal Medicine patients except for mortality, which was significantly higher in WE when stays were up to 48 h (*p* < 0.001), 72 h (*p* < 0.01) and 96 h (*p* < 0.05). For the hospital-wide patient group, these differences in mortality were only significantly higher (*p* < 0.01) when their stays did not exceed 48 h.

Multivariate analysis (Table 2) corroborated the significant association of mortality with admissions in WE, both in Internal Medicine patients and in the hospital as a whole. This association was stronger the shorter the length of stay, from an OR = 2.50 (95% CI 1.60–3.90) in Internal Medicine for stays of up to 48 h to an OR = 1.62 (95% CI 1.18–2.21) for stays of up to 96 h and losing significance for longer stays. This pattern was repeated for the hospital-wide group (Table 2) with an OR = 2.02 (95% CI 1.66–2.47) for stays up to 48 h and OR = 1.41 (95% CI 1.20–1.65) for stays up to 72 h.

**Table 2:** Eight logistic models are presented with death as the dependent variable: 4 for Internal Medicine patients according to whether their stay was 48, 72, 96, or more than 96 h; another 4 models for total hospital patients are displayed in the bottom panel of the table. OR (95% CI) are given for each variable.

Internal medicine patients		Stay of 48 h n = 879	Stay of 72 h n = 1409	Stay of 96 h n = 2008	Stay > 96 h n = 5648
Age		1.06 (1.04–1.08)	1.05 (1.03–1.06)	1.05 (1.03–1.06)	1.03 (1.02–1.04)
Male		1.98 (1.26–3.12)	1.56 (1.08–2.24)	1.49 (1.08–2.06)	1.21 (0.96–1.51)
Charlson		1.10 (1.01- 1.21)	1.15 (1.07–1.24)	1.17 (1.10–1.25)	1.17 (1.12–1.22)
Sepsis		5.52 (3.04–10.05)	5.18 (3.15–8.52)	5.20 (3.35–8.09)	1.35 (0.95–1.91)
Heart failure		1.31 (0.79–2.15)	1.15 (0.76–1.72)	0.96 (0.66–1.38)	0.97 (0.76–1.24)
Pneumonia		0.78 (0.36–1.70)	1.38 (0.81–2.34)	1.41 (0.88–2.25)	1.13 (0.79–1.60)
Year of discharge	2015	1	1	1	1
	2016	2.07 (0.66- 6.46)	2.13 (0.80–5.68)	1.56 (0.69- 3.52)	0.70 (0.47–1.04)
	2017	1.02 (0.36–2.90)	1.74 (0.70–4.34)	1.53 (0.72–3.27)	1.13 (0.80–1.61)
	2018	2.44 (0.87–6.88)	3.85 (1.56–9.51)	3.31 (1.56–7.03)	0.94 (0.65–1.35)
	2019	1.71 (0.62–4.69)	3.46 (1.43–8.35)	3.29 (1.59–6.80)	1.55 (1.10–2.18)
WE admission		**2.50 (1.60–3.90)**	**1.89 (1.33–2.70)**	**1.62 (1.18–2.21)**	**0.94 (0.75–1.17)**

Hospital-wide patients		Stay of 48 h n = 18,198	Stay of 72 h n = 27,342	Stay of 96 h n = 34,388	Stay > 96 h n = 48,758
Age		1.06 (1.06–1.07)	1.06 (1.06–1.07)	1.06 (1.06–1.06)	1.04 (1.03–1.04)
Male		1.88 (1.56–2.26)	1.76(1.51–2.05)	1.60 (1.40–1.84)	1.28 (1.17–1.39)
Severity upon admission	1	1	1	1	1
	2	3.94 (2.82–5.49)	3.57 (2.69–4.74)	3.06 (2.39–3.95)	1.34 (0.97–1.32)
	3	22.77 (16.53–31.38)	17.31 (13.21–22.68)	14.19 (11.16–18.05)	4.33 3.70–5.07)
	4	144.12 (94.93–218.80)	122.90 (86.52–174.58)	106.79 (78.74–144.84)	15.83 (13.34–18.80)
Year of discharge	2015	1	1	1	1
	2016	1.55 (1.08–2.21)	1.41 (1.04–1.91)	1.32 (1.00–1.73)	1.13 (0.97–1.32)
	2017	0.71 (0.52–0.99)	0.94 (0.71–1.24)	0.96 (0.75–1.23)	1.11 (0.96–1.27)
	2018	1.27 (0.92–1.75)	1.49 (1.14–1.96)	1.51 (1.19–1.91)	1.21 (1.05–1.38)
	2019	1.21 (0.88–1.67)	1.46 (1.12–1.92)	1.43 (1.12–1.81)	1.38 (1.21–1.58)
WE admission		**2.02 (1.66–2.47)**	**1.41 (1.20–1.65)**	**1.13 (0.98–1.30)**	**0.87 (0.80–0.95)**

In bold the highest OR for “Time to assesment”.

When the risk of death associated with admission on each day of the weekend was analysed separately (Table 3), we found that Internal Medicine patients admitted on Fridays and with stays of up to 48 h had an OR = 3.92 (95% CI 2.06–7.48) which decreased on Saturdays to OR = 2.38 (95% CI 1.14–4.99) and was not significant on Sundays (OR = 1.98; 95% CI 0.96–4.09). The same was true for patients in the whole hospital, where the risk of death was diluted from Friday to Sunday; also, the risk associated with admission on each day decreased with increasing length of stay (Table 4).

**Table 3:** Eight logistic models with death as dependent variable are presented, 4 for Internal Medicine patients and 4 for total hospital patients.

Internal medicine patients		Stay of 48 h n = 879	Stay of 72 h n = 1409	Stay of 96 h n = 2008	Stay > 96 h n = 5648
Age		1.06 (1.04–1.08)	1.05 (1.03–1.06)	1.05 (1.03–1.06)	1.03 (1.02–1.04)
Male		2.02 (1.28–3.19)	1.57 (1.09–2.26)	1.51 (1.09–2.09)	1.21 (0.96–1.51)
Charlson		1.10 (1.00–1.21)	1.15 (1.07–1.24)	1.17 (1.10–1.24)	1.17 (1.12–1.22)
Sepsis		5.75 (3.15–10.48)	5.23 (3.18–8.62)	5.25 (3.37–8.17)	1.34 (0.95–1.90)
Heart failure		1.28 (0.78–2.11)	1.11 (0.74–1.67)	0.95 (0.66–1.37)	0.97 (0.76–1.24)
Pneumonia		0.79 (0.37–1.71)	1.41 (0.82–2.40)	1.42 (0.88–2.27)	1.12 (0.79–1.60)
Year of discharge	2015	1	1	1	1
	2016	2.14 (0.69–6.71)	2.23 (0.83–5.97)	1.60 (0.70–3. 62)	0.70 (0.47–1.04)
	2017	0.98 (0.34–2.80)	1.76 (0.70–4.39)	1.59 (0.74–3.41)	1.12 (0.79–1.60)
	2018	2.31 (0.82–6.55)	3.84 (1.56–4.07)	3.38 (1.59–7.19)	0.93 (0.64–1.34)
	2019	1.65 (0.60–4.55)	3.42 (1.41–8.30)	3.33 (1.61–6.92)	1.55 (1.10–2.18)
Admission	Friday	**3.92 (2.06–7.48)**	**2.52 (1.57–4.07)**	**2.13 (1.41–3.23)**	0.90 (0.67–1.22)
	Saturday	**2.38 (1.14–4.99)**	**1.97 (1.12–3.45)**	**1.43 (0.89–2.31)**	0.99 (0.71–1.38)
	Sunday	1.98 (0.96–4.09)	1.23 (0.67–2.26)	1.31 (0.77–2.22)	1.06 (0.72–1.55)

Hospital-Wide Patients		Stay of 48 h n = 18,198	Stay of 72 h n = 27,342	Stay of 96 h n = 34,388	Stay > 96 h n = 48,758
Age		1.06 (1.06–1.07)	1.06 (1.06–1.07)	1.06 (1.06–1.06)	1.04 (1.03–1.04)
Male		1.88 (1.56–2.27)	1.78 (1.51–2.06)	1.61 (1.40–1.84)	1.28 (1.17–1.39)
Severity upon admission	1	1	1	1	1
	2	4.00 (2.87–5.57)	3.59 (2.71–4.77)	3.07 (2.39–3.96)	1.34 (1.13–1.61)
	3	22.84 (16.56–31.49)	17.26 (13.17–22.62)	14.16 (11.14–18.01)	4.33 (3.70–5.07)
	4	143.64 (94.58–218.17)	122.61 (86.26–174.29)	106.91(78.80–145.05)	15.85 (13.34–18.82)
Year of discharge	2015	1	1	1	1
	2016	1.54 (1.08–2.20)	1.40 (1.03–1.91)	1.31 (1.00–1.72)	1.13 (0.97–1.32)
	2017	0.71 (0.52–0.99)	0.94 (0.72–1.24)	0.96 (0.76–1.23)	1.11 (0.96–1.38)
	2018	1.26 (0.92–1.73)	1.49 (1.14- 1.95)	1.51 (1.19–1.91)	1.21 (1.05–1.38)
	2019	1.21 (1.88–1.67)	1.46 (1.12- 1.92)	1.42 (1.12–1.80)	1.38 (1.21–1.58)
Admission:	Friday	**2.93 (2.20–3.90)**	**1.88 (1.51- 2.35)**	**1.39 (1.15–1.68)**	0.89 (0.80–1.00)
	Saturday	**2.17 (1.61–2.94)**	**1.51 (1.18–1.93)**	1.13 (0.91–1.40)	0.81 (0.70–0.93)
	Sunday	1.22 (0.88–1.70)	0.89 (0.68–1.17)	0.84 (0.67–1.06)	0.90 (0.78–1.05)

One model was fitted for each stay stratum: up to 48, 72, 96, or more than 96 h. Weekend admission was categorised into Friday, Saturday and Sunday. For each variable, OR (95% CI) is given.

In bold the highest OR for “Time to assesment”.

**Table 4:** Eight logistic models with death as dependent variable are presented, 4 for Internal Medicine patients and 4 for total hospital patients.

Internal medicine patients		Stay of 48 hoursn = 879	Stay of 72 h n = 1409	Stay of 96 h n = 2008	Stay > 96 h n = 5648
Age		1.06 (1.03–1.08)	1.04 (1.03–1.06)	1.05 (1.03–1.06)	1.03 (1.02–1.04)
Male		2.09 (1.31–3.31)	1.60 (1.11–2.31)	1.52 (1.10–2.11)	1.21 (0.96–1.51)
Charlson		1.11 (1.01–1.22)	1.15 (1.07–1.24)	1.17 (1.10–1.25)	1.17 (1.12–1.22)
Sepsis		5.67 (3.10–10.38)	5.18 (3.14–8.55)	5.20 (3.34–8.10)	1.34–0.95–1.90)
Heart failure		1.27 (0.77–2.09)	1.11(0.74–1.68)	0.95 (0.66–1.37)	0.97 (0.76–1.24)
Pneumonia		0.75 (0.34–1.65)	1.34 (0.78–2.32)	1.39(0.86–2.24)	1.12 (0.79–1.60)
Year of discharge	2015	1	1	1	1
	2016	2.11(0.67–6.60)	2.35 (0.86–6.44)	1.60 (0.70–3.65)	0.69 (0.46–1.03)
	2017	0.91 (0.32–2.61)	1.83 (0.72–4.68)	1.58 (0.74–3.41)	1.12 (0.79–1.60)
	2018	2.21 (0.78–6.28)	4.05 (1.60–10.27)	3.42 (1.60–7.31)	0.93 (0.64–1.34)
	2019	1.55 (0.56–4.28)	3.50 (1.41–8.70)	3.31 (1.59–6.89)	1.55 (1.10–2.18)
Time to assessment	Delay 1 d	1.49 (0.70–3.16)	1.02 (0.55–1.90)	1.12 (0.65–1.91)	1.05 (0.73–1.50)
	Delay 2 d	**2.40 (1.24–4.65)**	**1.92 (1.15–3.21)**	1.43 (0.91–2.25)	0.98 (0.72–1.35)
	Delay 3 d	NA	**2.56 (1.58–4.17)**	**2.11 (1.39–3.21)**	0.88 (0.65–1.19)
	Delay 4 d	NA	NA	**5.51 (1.42–21.40)**	0.62 (0.22–1.72)
	Delay 5 d	NA	NA	NA	0. 50 (0.07–3.72)

Hospital-wide patients		Stay of 48 h n = 18,198	Stay of 72 h n = 27,342	Stay of 96 h n = 34,388	Stay > 96 h n = 48,758
Age		1.06 (1.06–1.07)	1.08 (1.08–1.09)	1.06 (1.05–1.06)	1.04 (1.03–1.04)
Male		1.93 (1.57–2.36)	1.77 (1.51–2.08)	1.61 (1.40 v 1.85)	1.28 (1.17–1.39)
Severity upon admission	1	1	1	1	1
	2	3.63 (2.52–5.25)	3.58 (2.68–4.76)	5.01 (3.58–7.00)	1.34 (1.13–1.60)
	3	20.90 (14.70–29.70)	17.48 (13.28–22.99)	23.25 (16.76–32.24)	4.33 (3.69–5.07)
	4	137.78 (87.74–216.36)	125.31 (87.74–178.99)	174.07(119.34–253.90)	15.83(13.33–18.80)
Year of discharge	2015	1	1	1	1
	2016	1.45 (0.99–2.14)	1.37 (1.01–1.87)	0.94 (0.70–1.27)	1.13 (0.97–1.32)
	2017	0.67 (0.47–0.94)	0.90 (0.68–1.18)	0.96 (0.75–1.22)	1.11 (0.96–1.28)
	2018	1.18 (1.83–1.66)	1.49 (1.13–1.95)	1.50 (1.18–1.91)	1.21 (1.05–1.38)
	2019	1.03 (0.73–1.45)	1.41 (1.08–1.85)	1.38 (1.09–1.76)	1.38 (1.21–1.59)
Time to assessment	Delay 1 d	1.12 (0.81–1.54)	0.84 (0.65–1.10)	0.80 (0.62–1.00)	0.94 (0.81–1.08)
	Delay 2 d	**1.95 (1.47–2.59)**	**1.47 (1.17–1.85)**	1.15 (0.94–1.42)	0.86 (0.76–0.98)
	Delay 3 d	NA	**1.96 (1.56–2.45)**	**1.45 (1.19–1.76)**	0.89 (0.79–1.00)
	Delay 4 d	NA	NA	**2.94 (1.58–5.48)**	1.00 (0.71–1.43)
	Delay 5 d	NA	NA	NA	0.88 (0.47–1.66)

One model was fitted for each stratum of stay: up to 48, 72, 96, or more than 96 h. Instead of weekend admission, the time elapsed (days) until the patient was assessed by the physicians of the department to which he/she was admitted (not by the on-call physicians) was included as an independent variable. OR (95% CI) are given for each variable.

NA: not applicable. In bold the highest OR for “Time to assesment”.

Table 4 presents the logistic analyses in which the day of admission was not included but instead the time it took for patients to be seen by the physicians in the department to which they were admitted. It shows that Internal Medicine patients with stays of up to 48 h suffered significant increases in the risk of death when the delay reached 2 days (OR = 2.40; 1.24–4.65) and reached risks of 5.51 (1.42–21.40) when the delay was 4 days; again, the risk associated with each day of delay decreased with increasing length of stay. The same pattern held for hospital-wide patients, who reached risks of 2.94 (1.58–5.48) for 4-day delays and 96-h stays. Supplementary Table 5 shows that there was no relevant change when the logistic adjustment for all hospital patients was repeated after excluding admissions for delivery and caesarean section (n = 14,208); this analysis was also repeated by adjusting Cox proportional risk models (supplementary Table 6) to corroborate the increase of the risk of death in a dose–response manner, reaching HR = 2.03 (1.16–3.55) when the delay was 2 days in IM patients with stays of up to 48 h, and HR = 2.74 (1.00–7.54) when the delay was 4 days.

**Figure 1:**
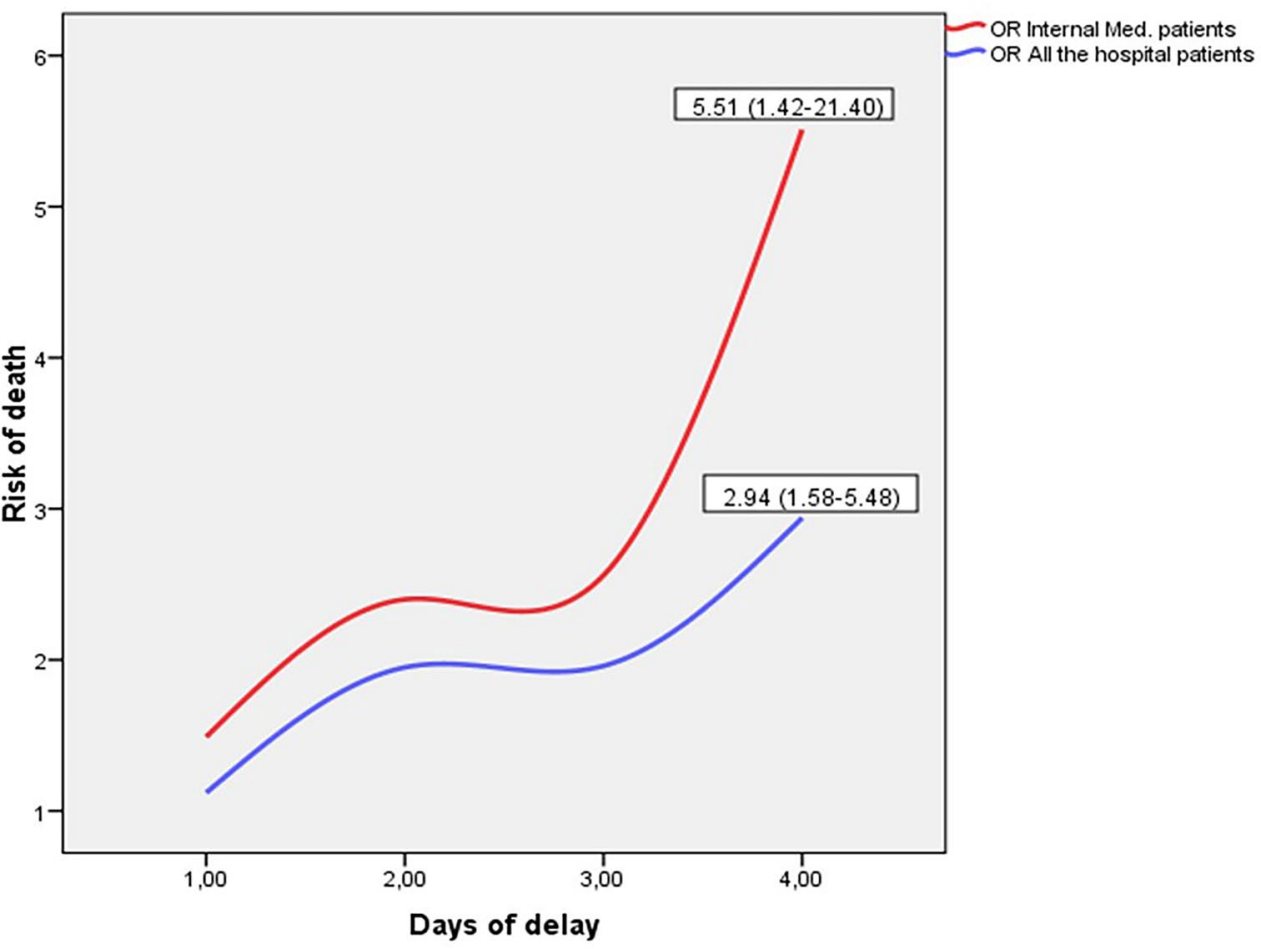
Evolution of the risk of death (OR for WE/WD) according to the time elapsed until the patient's medical assessment.

Figure 1 represents the evolution of the risk of death after admission on a WE compared to admission on a WD, both in Internal Medicine patients and in the hospital as a whole, according to the time elapsed from admission until the patient was assessed by the physicians of his or her department.

## Discussion

This study corroborates that patients admitted to hospital for urgent problems during WE suffer a higher risk of death than those admitted on WD. The study also shows that the factor with the greatest effect on this increased risk of death is the time that elapses from admission by the physicians on duty until the patient's care is taken over by the physicians of the department to which the patient was admitted; as a result, Friday is the day with the highest risk of death after admission and, in contrast, Sunday has no weekend effect.

Although this problem has been extensively studied, there is a lack of evidence on what happens to Internal Medicine patients, usually with multiple serious conditions. Among the few articles analysing Internal Medicine patients is that of Marco et al.^4^, a multicentre study conducted with a large sample size that found that mortality at 48 h of admission increased by up to 60% on weekends in all hospitals in the country. In our case, we also analysed other longer lengths of stay, and found that the WE effect was no longer significant after the fifth day of admission. The importance of the first days of admission has been widely described in patients with various diagnoses admitted to different hospital services^3,4,23,38^, and it has been described that the risk of death after admission decreases by 1% for each additional day of stay^23^. We have found that the main factor behind this effect is the time elapsed from emergency admission to the start of normal hospital care, corroborated by differentiating between weekend days and finding that patients admitted on Sunday do not suffer this effect as they receive normal care the following day. It is logical to think that doctors who admit a patient at 3 in the morning do not have such a clear mind and can make mistakes, however, in WD the admitted patient will be evaluated after a few hours by a doctor with a clear head who can fix diagnostic errors or omissions of treatment as a result of the fatigue of the doctor on duty; by contrast, when admitted on the WE these possible errors will take time to be detected since the inpatient will not be evaluated again until Monday. The same results were reproduced when we analysed the base that included all the patients in the hospital, with somewhat attenuated risks with respect to the Internal Medicine patients, perhaps because the heterogeneity of diagnoses was much greater in these patients; and the same occurred again after excluding patients admitted for childbirth and caesarean section, which we did because they are a very important group (17% of the total) with a specific profile of sex, young age, low mortality and short stay.

There are studies whose patients had an increased risk of death after admission over a WE and this effect was reduced by opening a medical admissions unit^39^, while other studies found a reduction of mortality after providing enhanced WE staffing for acute medical inpatient services^40^. It has been pointed out that the excess mortality patterns of the WE vary widely for different diagnostic groups, and recognising these patterns should help to minimise the excess mortality^41^. However the adoption of seven day clinical standards in the delivery of emergency hospital services did not reduce the WE effect^42^, and the authors of other investigations conclude that further work is required to examine non evaluated potential explanations for the WE such as staffing levels and other organisational factors^7^. Some important outputs suggest that the relationship between consultant presence and mortality is tenuous at best, and that causal pathways for the WE effect may extend into the prehospital setting, in the community that precede hospital admission^43,44^. Our study proposes to work on the time elapsed from emergency admission to the start of normal hospital care.

The importance for patient prognosis of prompt attention is evident, as is the availability of the hospital's full diagnostic and therapeutic capacity, which is not fully available during the WE periods. The problem we have studied is not diagnosis or treatment in the emergency services, whose analysis would require a different design. What we have studied is the hospital mortality of patients once admitted, that is, mortality related to the care received during their stay in the beds of the different specialties. This is compounded by another factor that suggests that the problem may worsen over the years: given the progress of medicine, the increasing prevalence of multiple simultaneous chronic diseases and demographic ageing, Internal Medicine services in Spain have gone from 460,000 discharges in 2005 to almost 750,000 in 2019^45^.

The patients in our study were those admitted through the emergency department. In our environment (Canary Islands), palliative care only exists as hospital units and it has the same accessibility on WD and WE; unfortunately, patients end up dying in the hospital regardless of the day of the week. The results of the group of patients admitted to Internal Medicine, excluding those who were moved to the palliative care unit, were similar to the results in the hospital-wide patients.

Respiratory infections, heart failure and sepsis are the most frequent discharge diagnoses in Internal Medicine^28,46^. For this reason, these are the diseases that we have adjusted for in our analysis. It is noteworthy that the diagnosis of sepsis had a high mortality, with a threefold increase in the risk of death in the first days of stay in our centre. However, in 2020, the so-called "sepsis code" was introduced in our hospital, an alarm code for early assessment and follow-up in the ICU, so these figures may have improved with respect to the results prior to that year.

The main limitations of our study are related to the retrospective design and the use of an administrative database. We could not analyse many factors: interindividual differences among on-call doctors, proportion of misdiagnoses in the ER, mortality variability during periods of high care pressure, or differentiate whether the death of a patient admitted to IM occurred in an ER bed or in a department bed; in addition, in an administrative database there is always a risk of coding errors as severity and comorbidities were not registered for the purpose of the study. However, the quality of the MBDS is proven and many rigorous studies with its data have been validated, and our findings are consistent with the well-known ‘weekend-effect’ and the ‘dose–response relationship’ (the longer it takes before the regular teams sees the patient, the higher the chance of dying) is an extra proof of validity of our results. Another limitation of our study can be related to not analysing data relating to the medical personnel present every day in the hospital; although we describe in the Methods section the composition of the on-call medical team, it is not available the composition of the staff of each medical specialty along the studied period. One more limitation of the study is that it is a single-centre study and limited to 5 years, although the patient samples we studied for both Internal Medicine and the hospital-wide group are very large and give consistency to the data; on the other hand, as Tenerife is an island it represents a closed eco-system of care, which is useful in the context of resource utilisation on WD vs WE.

## Conclusion

Whether it was IM patients or hospital-wide patients, hospital admission on WE increases mortality in patients with short stays, and the main factor associated with this is the time that elapses from admission until the patient's care is taken over by the department to which he/she was admitted. Therefore, there is a ‘dose–response relationship’ where Friday is the day with the highest risk of death after admission and, in contrast, Sunday has no weekend effect. Hospitals must implement measures that guarantee that patients admitted during the weekend are treated within a similar time frame as those admitted during weekdays.

### Supplementary Information

Supplementary Information.

## Data Availability

The data, the Bioethics Committee approval and the analysis plan that support the findings of this study are available on request from the corresponding author.
