# Supplementary material for: The dose–response effect of time between emergency admission and inpatient care on mortality
Source: Sci Rep. 2023 Dec 14;13:22244. doi: 10.1038/s41598-023-49090-5 (PMC10721600; doi:10.1038/s41598-023-49090-5)
Supplement: Supplementary file 1 — Supplementary Information. [file 41598_2023_49090_MOESM1_ESM.docx]

**An explanation of how our hospital operates:**

The on-call medical team overseeing emergency care during the evening and night hours, as well as on non-working days, personally cared for patients, without consultants on duty.

The degree of supervision of doctors in training depends on their training time, varying between permanent supervision in their first year to only supervision of doubts in their 4th-5th year.

During the weekend there is no daily visit to the inpatients, but only the on-call team; Except for a call from the nurse, patients admitted are not assessed until Monday.

On-call doctors can contact doctors from other specialties to discuss diagnostic or therapeutic questions.

During the studied period (2015-2019) there was no MET/RRT system (rapid response team for unstable patients). Starting in 2020, an extended intensive care unit was established, a program with various clinical-analytical data that raises alarm bells for seriously ill patients to be evaluated.

In the medical area (Internal Medicine, Geriatrics, Digestive, Endocrinology, Rheumatology and Medical Oncology) it is structured as follows: During morning hours, each doctor is responsible for 8 to 10 patients a day: ratio 1/10. During on-call hours, all admitted patients (about 200 patients) are under the care of the on-call doctors (2 assistants and 3 residents), to cover possible incidents or complications. In addition, calls from surgical services in case of medical complications are also assumed. Likewise, the guard is responsible for admitting patients to the emergency room (about 15 daily assessments).

In the HUNSC, patients are not admitted directly by the emergency department doctors, who carry out the first evaluation and, if they consider the patient a candidate for admission into a hospital service, they notify the specialist doctor of that service. This doctor is responsible for deciding admission after taking an exhaustive medical history, establishing treatment, and informing the family.

**Supplementary table 1.** Association of the length of stay with the severity on admission in hospital-wide patients and in the deceased patients. Also, association of the length of stay with mortality.

| Hospital-wide patients | | P* | Deceased  patients | | P* | Exitus | Lenght of stay | P* |
| --- | --- | --- | --- | --- | --- | --- | --- | --- |
| Severity upon admission | Lenght of stay |  | Severity upon admission | Lenght of stay |  |  |  |  |
| 1 | 6.8±11.1 | <0.001 | 1 | 12.4±14.2 | <0.001 | No | 9.8±17.4 | <0.001 |
| 2 | 9.4±15.0 |  | 2 | 12.5±28.1 |  | Yes | 15.2±24.4 |  |
| 3 | 13.7±18.1 |  | 3 | 14.5±19.7 |  |  |  |  |
| 4 | 26.1±50.7 |  | 4 | 20.4±34.8 |  |  |  |  |

*Non parametric Kruskal-Wallis test.

**Supplementary table 2.** Distribution of patients admitted to the Internal Medicine department and to the hospital-wide group according to days of the week.

|  | PATIENTS | |
| --- | --- | --- |
|  | Internal Medicine | Hospital-wide |
| Monday | 1027 (13,4) | 12553 (15,1) |
| Tuesday | 1134 (14,8) | 13010 (15,6) |
| Wednesday | 1271 (16,6) | 13462 (16,2) |
| Thursday | 1244 (16,2) | 13216 (15,9) |
| Friday | 1320 (17,2) | 13040 (15,7) |
| Saturday | 946 (12,4) | 9423 (11,3) |
| Sunday | 714 (9,3) | 8442 (10,2) |
| Total | 7656 (100,0) | 83146 (100,0) |

**Supplementary table 3.** List of the 10 most common diagnoses in WD and WE emergency admissions.

| Working days | Code | Admissions  % |
| --- | --- | --- |
| Pneumonia | J189 | 1.5 |
| Urinary tract infection | N390 | 1.4 |
| Heart failure | I509 | 1.4 |
| Chronic obstructive pulmonary disease with acute exacerbation | J441 | 1.1 |
| Acute non-ST elevation myocardial infarction | I214 | 1.1 |
| Hypertensive chronic heart and kidney disease with heart failure and chronic kidney disease stages 1 to 4 | I130 | 1.0 |
| Acute lower respiratory tract infection | J22 | 1.0 |
| Hypertensive heart disease with heart failure | I110 | 0.9 |
| Pneumonitis due to food inhalation and vomiting | J690 | 0.9 |
| Other respiratory disorders | J988 | 0.7 |

| Weekends | Code | Admissions  % |
| --- | --- | --- |
| Pneumonia | J189 | 1.6 |
| Urinary tract infection | N390 | 1.5 |
| Heart failure | I509 | 1.5 |
| Acute non-ST elevation myocardial infarction | I214 | 1.4 |
| Chronic obstructive pulmonary disease with acute exacerbation | J441 | 1.3 |
| Hypertensive chronic heart and kidney disease with heart failure and chronic kidney disease stages 1 to 4 | I130 | 1.0 |
| Acute lower respiratory tract infection | J22 | 1.0 |
| Hypertensive heart disease with heart failure | I110 | 1.0 |
| Pneumonitis due to food inhalation and vomiting | J690 | 0.9 |
| Acute appendicitis | K3580 | 0.8 |

**Supplementary table 4.** Comparison of patients, and their mortality, with respect to weekday (WD) or weekend (WE) admission according to length of stay.

| **INTERNAL MEDICINE PATIENTS** | | Stay ≤ 48 hours | | p | Stay ≤ 72 hours | | p | Stay ≤ 96 hours | | p | Stay > 96 hours | | p |
| --- | --- | --- | --- | --- | --- | --- | --- | --- | --- | --- | --- | --- | --- |
|  |  | WD (%)  n=635 | WE (%)  n=244 |  | WD (%)  n=904 | WE (%)  n=505 |  | WD (%)  n=1187 | WE (%)  n=821 |  | WD (%)  n=3109 | WE (%)  n=2539 |  |
| Age x±SD | | 71.0 ±16.9 | 71.5 ± 18.7 | NS | 70.0 ±17.3 | 69.4 ±19.2 | NS | 69.6 ±17.4 | 69.1 ±18.7 | NS | 69.8±15.3 | 70.0±15.3 | NS |
| Men | | 315 (49.6) | 122 (50.0) | NS | 455 (50.3) | 254 (50.3) | NS | 597 (50.3) | 417 (50.8) | NS | 1678 (54.0) | 1400 (55.1) | NS |
| Charlson x±SD | | 2.6 ±2.6 | 2.7 ±2.5 | NS | 2.6 ±2.6 | 2.6 ±2.5 | NS | 2.6 ±2.6 | 2.5 ±2.5 | NS | 2.7±2.4 | 2.5±2.4 | NS |
| Sepsis | | 62 (9.8) | 23 (9.4) | NS | 79 (8.7) | 41 (8.1) | NS | 100 (8.4) | 65 (7.9) | NS | 293 (9.4) | 273(10.8) | NS |
| Heart failure | | 156 (24.6) | 59 (24.2) | NS | 221 (24.4) | 118 (23.4) | NS | 296 (24.9) | 182 (22.2) | NS | 837 (26.9) | 643 (25.3) | NS |
| Pneumonia | | 60 (9.4) | 15 (6.1) | NS | 78 (8.6) | 42 (8.3) | NS | 94 (7.9) | 72 (8.8) | NS | 280 (9.0) | 224 (8.8) | NS |
| Year of discharge | 2015 | 84 (13.2) | 21 (8.6) | <0.01 | 131 (14.5) | 68 (13.5) | <0.01 | 173 (14.6) | 126 (15.3) | <0.01 | 681 (21.9) | 428 (16.9) | <0.01 |
|  | 2016 | 74 (11.7) | 27 (11.1) |  | 117 (12.9) | 77 (15.2) |  | 177 (14.9) | 139 (16.9) |  | 585 (18.8) | 472 (18.6) |  |
|  | 2017 | 159 (25.0) | 85 (34.8) |  | 214 (23.7) | 150 (29.7) |  | 279 (23.5) | 230 (28.0) |  | 629 (20.2) | 592 (23.3) |  |
|  | 2018 | 106 (16.7) | 51 (20.9) |  | 156 (17.3) | 99 (19.6) |  | 202 (17.0) | 145 (17.7) |  | 642 (20.6) | 546 (21.5) |  |
|  | 2019 | 212 (33.4) | 60 (24.6) |  | 286 (31.6) | 111 (22.0) |  | 356 (30.0) | 181 (22.0) |  | 572 (18.4) | 501 (19.7) |  |
| Mortality | | **61 (9.6)** | **49 (20.1)** | **<0.001** | **84 (9.3)** | **75 (14.9)** | **<0.01** | **99 (8.3)** | **95 (11.6)** | **<0.05** | 202 (6.5) | 156 (6.1) | NS |
| **HOSPITAL-WIDE PATIENTS** | | Stay ≤ 48 hours | | p | Stay ≤ 72 hours | | p | Stay ≤ 96 hours | | p | Stay > 96 hours | | p |
|  |  | WD (%)  n=12028 | WE (%)  n=6872 |  | WD (%)  n=16334 | WE (%)  n=11008 |  | WD (%)  n=20112 | WE (%)  n=14276 |  | WD (%)  n=28159 | WE (%)  n=20599 |  |
| Age x±SD | | 46.7±20.5 | 43.2±19.6 | <0.001 | 47.6±20.8 | 44.3±20.1 | <0.001 | 48.2±20.9 | 46.8±20.9 | <0.001 | 63.3±18.8 | 64.5±18.8 | <0.001 |
| Men | | 3768 (31.3) | 1978 (28.8) | <0.001 | 5254 (32.2) | 3182 (28.9) | <0.001 | 6603 (32.8) | 4542 (31.8) | <0.05 | 14026 (49.8) | 10669 (51.8) | <0.001 |
| Year of discharge | 2015 | 2480 (20.6) | 1277 (18.6) | <0.001 | 3259 (20.0) | 1988 (18.1) | <0.001 | 3922 (19.5) | 2553 (17.9) | <0.01 | 5448 (19.3) | 3330 (16.2) | <0.001 |
|  | 2016 | 2280 (19.0) | 1321 (19.2) |  | 3083 (18.3) | 2139 (19.4) |  | 3822 (19.0) | 2750 (19.3) |  | 5204 (18.5) | 3761 (18.3) |  |
|  | 2017 | 2605 (21.7) | 1551 (22.6) |  | 3552 (21.7) | 2449 (22.2) |  | 4433 (22.0) | 3155 (22.1) |  | 5742 (20.4) | 4401 (21.4) |  |
|  | 2018 | 2345 (19.5) | 1452 (21.1) |  | 3223 (19.7) | 2309 (21.0) |  | 3957 (19.7) | 3006 (21.1) |  | 6032 (21.4) | 4592 (22.3) |  |
|  | 2019 | 2318 (19.3) | 1271 (18.5) |  | 3217 (19.7) | 2123 (19.3) |  | 3978 (19.8) | 2812 (19.7) |  | 5733 (20.4) | 4515 (21.9) |  |
| Mortality | | **408 (3.4)** | **294 (4.3)** | **<0.01** | 567 (3.5) | 404 (3.7) | NS | 684 (3.4) | 509 (3.6) | NS | 1492 (5.3) | 1031 (5.0) | NS |

| **HOSPITAL-WIDE PATIENTS**  **EXCEPT. CHILDBIRTH/ C. SECTION** | | **Stay of**  **48 hours**  n=11627 | **Stay of**  **72 hours**  n=16847 | **Stay of**  **96 hours**  n=22092 | **Stay > 96 hours**  n=46846 |
| --- | --- | --- | --- | --- | --- |
| Time to assessment | Delay 1 d. | 0.95 (0.72 - 1.26) | **0.77 (0.61 - 0.98)** | 0.82 (0.67 - 1.01) | 0.95 (0.82 - 1.08) |
|  | Delay 2 d. | **1.88 (1.47- 2.40)** | **1.37 (1.11 - 1.68)** | 1.06 (0.88 - 1.27) | 0.87 (0.77 - 0.98) |
|  | Delay 3 d. | NA | **1.89 (1.54 - 2.29)** | **1.45 (1.22 - 1.72)** | 0.86 (0.76 - 0.96) |
|  | Delay 4 d. | NA | NA | **2.54 (1.55 - 4.16)** | 0.91 (0.64 - 1.28) |
|  | Delay 5 d. | NA | NA | NA | 0.81 (0.44 - 1.49) |

**Supplementary table 5.** Four logistic models with death as the dependent variable, adjusted for age, sex and year of discharge, are summarised for total hospital patients after excluding admissions for delivery or caesarean section. One model was fitted for each stay stratum: up to 48, 72, 96, or more than 96 hours. Instead of weekend or holiday admission, the time elapsed (days) until the patient was assessed by the physicians of the department where he/she was admitted (not by the on-call physicians) was included as an independent variable. OR (95% CI) are given.

NA: not applicable.

**Supplementary table 6.** Eight Cox regression models with death as the dependent variable, adjusted for age, sex and year of discharge, are summarised for total hospital patients after excluding admissions for delivery or caesarean section. One model was fitted for each stay stratum: up to 48, 72, 96, or more than 96 hours. Instead of weekend or holiday admission, the time elapsed (days) until the patient was assessed by the physicians of the department where he/she was admitted (not by the on-call physicians) was included as an independent variable. HR (95% CI) are given.

| **INTERNAL MEDICINE PATIENTS** | | **Stay of**  **48 hours**  n=879 | **Stay of**  **72 hours**  n=1409 | **Stay of**  **96 hours**  n=2008 | **Stay**  **> 96 hours**  n=5648 |
| --- | --- | --- | --- | --- | --- |
| Time to assessment | Delay 1 d. | 1.24 (0.65 – 2.38) | 0.81 (0.46 – 1.44) | 0.93 (0.57 - 1.52) | 0.97 (0.69 - 1.37) |
|  | Delay 2 d. | **2.03 (1.16 – 3.55)** | 1.38 (0.88 – 2.18) | 1.03 (0.69 – 1.57) | 0.99 (0.73 - 1.34) |
|  | Delay 3 d. | NA | **1.64 (1.08 – 2.49)** | **1.45 (1.00 – 2.09)** | 0.87 (0.65 – 1.17) |
|  | Delay 4 d. | NA | NA | **2.74 (1.00 – 7.54)** | 0.62 (0.23 - 1.67) |
|  | Delay 5 d. | NA | NA | NA | 0.59 (0.08 – 4.20) |
| **HOSPITAL-WIDE PATIENTS**  **EXCEPT. CHILDBIRTH/ C. SECTION** | | **Stay of**  **48 hours**  n=11627 | **Stay of**  **72 hours**  n=16847 | **Stay of**  **96 hours**  n=22092 | **Stay > 96 hours**  n=46846 |
| Time to assessment | Delay 1 d. | 1.51 (1.21 - 1.88) | **0.80 (0.64 – 1.00)** | 0.75 (0.61 – 0.91) | 01.02 (0.90 - 1.17) |
|  | Delay 2 d. | **2.54 (2.06- 3.14)** | **1.22 (1.01 - 1.47)** | 0.95 (0.80 - 1.13) | 0.95 (0.84 – 1.07) |
|  | Delay 3 d. | NA | **1.45 (1.21 – 1.72)** | **1.05 (0.90 - 1.23)** | 0.90 (0.81 – 1.01) |
|  | Delay 4 d. | NA | NA | **1.73 (1.13 – 2.64)** | 0.99 (0.71 - 1.37) |
|  | Delay 5 d. | NA | NA | NA | 0.94 (0.52 - 1.70) |

NA: not applicable.
